# Application of Near-Infrared Spectroscopy in Early Detection of Antidepressant Treatment Efficacy in Major Depressive Disorder: A Longitudinal Study

**DOI:** 10.62641/aep.v53i2.1708

**Published:** 2025-03-05

**Authors:** Jianjie Huang, Jianmin Shan

**Affiliations:** ^1^Mental Comprehensive Ward II, Wenzhou Seventh People’s Hospital, 325000 Wenzhou, Zhejiang, China; ^2^Severe Female Ward, Wenzhou Seventh People’s Hospital, 325000 Wenzhou, Zhejiang, China

**Keywords:** major depressive disorder, near-infrared spectroscopy, hemodynamics, cognitive tasks, clinical outcomes

## Abstract

**Background:**

Major depressive disorder (MDD) is a prevalent and debilitating mental health condition, necessitating early detection and effective treatment strategies. Near-infrared spectroscopy (NIRS) is a promising neuroimaging technique for monitoring cerebral hemodynamics and may serve as an objective biomarker for MDD diagnosis and treatment efficacy. This study aimed to investigate the utility of NIRS in the early detection and longitudinal monitoring of antidepressant treatment efficacy in MDD patients.

**Methods:**

This longitudinal study, conducted from May 2022 to May 2024, included 138 participants. After propensity score matching analyses, 80 were included, including 40 MDD patients and 40 healthy controls matched for age, gender, race, education, height, weight, and body mass index (BMI). Participants underwent NIRS measurements during cognitive tasks, including verbal fluency, sustained attention (e-primer), and one-back memory tests. Clinical assessments were conducted using the Hamilton Depression Scale (HAMD), Hamilton Anxiety Scale (HAMA), Clinical Global Impression (CGI), Continuous Performance Test (CPT), and one-back tests at baseline and after treatment at 4 weeks and 24 weeks. Statistical analyses were performed to evaluate changes in oxygenated hemoglobin (HbO) and deoxygenated hemoglobin (HbR) levels and their correlation with clinical outcomes.

**Results:**

At baseline, MDD patients had significantly lower HbO and higher HbR levels compared to controls (*p* < 0.01). After treatment, HbO increased (4.77 ± 1.23 to 5.37 ± 1.21 μmol/L, *p* < 0.05) while HbR decreased (3.46 ± 0.98 to 2.91 ± 0.96 μmol/L, *p* < 0.05) in the MDD group. However, these levels differed significantly from controls at 4 weeks (*p* < 0.01). By 24 weeks, HbO further increased (6.01 ± 1.08 μmol/L, *p* < 0.05), and HbR further decreased (2.19 ± 0.71 μmol/L, *p* < 0.05), with no significant differences from controls (*p* > 0.05). Clinically, MDD patients showed significant improvements in HAMD, HAMA, CGI, CPT, and one-back scores over 24 weeks (all *p* < 0.05). At 4 weeks, HAMD, HAMA, and CGI scores were higher, and CPT and one-back responses were lower than controls (*p* < 0.01). By 24 weeks, HAMD, HAMA, and CGI scores remained higher (*p* < 0.01), and CPT and one-back responses were lower than controls (*p* < 0.01).

**Conclusion:**

This study underscores the potential of NIRS as a non-invasive, objective tool for early detection and monitoring of treatment efficacy in MDD. The significant correlations between NIRS findings and clinical improvements highlight its utility in personalized treatment strategies, paving the way for more effective management of MDD.

## Introduction

Major depressive disorder (MDD) is a prevalent and debilitating psychiatric condition characterized by persistent feelings of sadness, loss of interest or pleasure in most activities, and various physical and cognitive symptoms [1, 2]. This disorder affects millions of people worldwide, contributing to the global burden of disease due to its chronic nature, potential for recurrence, and impact on functional impairment and quality of life [3]. According to the WHO, depression is a leading cause of disability, underscoring the significance of effective diagnosis and treatment strategies [4].

Early detection and effective management of MDD are crucial for improving patient outcomes. Traditional methods for diagnosing and monitoring the disorder rely primarily on self-reported symptoms and clinical assessments, which are subjective and vary significantly among patients [5]. Therefore, there is a growing interest in identifying objective biomarkers to aid in the early detection and monitoring of treatment efficacy in this condition. Among the numerous potential biomarkers, neuroimaging techniques are prominent because they provide direct insights into brain function and structure [6, 7].

Near-infrared spectroscopy (NIRS) is a non-invasive neuroimaging technique that measures changes in the concentration of oxygenated hemoglobin (HbO) and deoxygenated hemoglobin (HbR) in the brain [8, 9]. Utilizing near-infrared light, NIRS penetrates the scalp and skull, reaching cortical areas, thus allowing for the monitoring of cerebral hemodynamics [10]. HbO and HbR are crucial indicators of cerebral oxygenation and blood flow, indicating neuronal activity and neurovascular coupling [11]. In individuals with MDD, studies have shown altered HbO and HbR levels, mainly in the prefrontal cortex, which is associated with mood regulation, decision-making, and cognitive control [12, 13]. Reduced HbO levels indicate decreased neuronal activity and impaired neurovascular function, while elevated HbR levels suggest inefficient oxygen utilization, both contributing to the cognitive deficits and emotional dysregulation observed in MDD patients [12].

Near-infrared spectroscopy (NIRS) offers numerous advantages over other neuroimaging modalities, such as functional magnetic resonance imaging (fMRI) and positron emission tomography (PET), including portability, relatively low cost, and the ability to be used in naturalistic settings [14, 15]. These features make NIRS suitable for longitudinal studies and clinical settings where frequent monitoring is required. The application of NIRS in psychiatry has shown promise in detecting abnormalities in brain function associated with various psychiatric disorders, including schizophrenia, bipolar disorder, and MDD [16, 17]. Previous studies have demonstrated that individuals with MDD exhibit altered hemodynamic responses in the prefrontal cortex, a brain region implicated in mood regulation, decision-making, and cognitive control [18, 19]. These abnormalities in cerebral oxygenation and blood flow can be detected by NIRS, providing potential biomarkers for diagnosis and treatment monitoring [20, 21].

A critical challenge in the treatment of MDD is the variability in patient responses to antidepressant medications [22]. Selective serotonin reuptake inhibitors (SSRIs) are commonly prescribed for MDD. They are generally well-tolerated, with a lower risk of severe side effects compared to other classes of antidepressants, making them suitable for long-term treatment in a diverse patient population [23]. Identifying early indicators of treatment efficacy helps clinicians tailor treatment plans more effectively, reducing the trial-and-error period and improving overall treatment outcomes [24]. NIRS serves as an early indicator of treatment response by detecting changes in cerebral hemodynamics that precede clinical improvements. While previous studies have demonstrated that individuals with MDD exhibit altered hemodynamic responses in the prefrontal cortex [25, 26], the use of NIRS as a longitudinal monitoring tool for antidepressant treatment efficacy remains underexplored. Numerous studies focus on cross-sectional data, limiting the understanding of dynamic changes in brain hemodynamics over the course of treatment [14, 27].

The present longitudinal study aimed to investigate the utility of NIRS in the early detection and monitoring of antidepressant treatment efficacy in patients with MDD. The primary objectives are to establish normative NIRS spectra for patients at baseline, monitor changes in HbO and HbR levels during cognitive tasks over the treatment period, and correlate these changes with clinical outcomes measured by standard psychiatric scales. The study focused on the prefrontal cortex to elucidate the relationship between cerebral hemodynamics and antidepressant treatment efficacy. Including cognitive tasks such as verbal fluency, sustained attention, and one-back memory tests during NIRS measurements allowed for a comprehensive assessment of functional brain activity [28]. These tasks were designed to engage the prefrontal cortex, providing a robust framework for detecting hemodynamic changes associated with MDD and its treatment.

In summary, this study contributes to the growing body of evidence supporting NIRS as a non-invasive, objective tool for early detection and monitoring of treatment efficacy in MDD. The findings from the study provide insights into the neurobiological mechanisms underlying MDD and its response to treatment, paving the way for more personalized and effective therapeutic strategies for patients suffering from this debilitating disorder.

## Materials and Methods

### Participants

A total of 138 participants who diagnosed with MDD according to the Diagnostic and Statistical Manual of Mental Disorders (DSM-IV, Fourth Edition) [29] criteria as well as who had health checkups who attended the Wenzhou Seventh People’s Hospital from May 2022 to May 2024 were collected. As there were significant differences in age and gender between the two groups (*p* < 0.01, **Supplementary Table 1**), we performed propensity score matching (PSM) analyses. For the PSM analyses, we used a matching criterion with a caliper value of 0.2, which means that we matched the samples with the requirement that the difference in propensity scores between the matched pairs did not exceed 0.2. By doing so, we screened 40 samples from each group out of a total of 138 samples, for a total of 80 samples, ensuring that the two groups were balanced on the key covariates.

Patients were selected based on the following criteria: a Hamilton Depression Scale (HAMD) score of 17 or higher [30], no prior antidepressant medication or a medication washout period of at least two weeks, between 18 and 60 years old, a minimum of junior high school education, and right-handedness. Exclusion criteria included intellectual disability, organic brain disease, pregnancy or lactation, a history of hypomanic, manic, or mixed episodes, significant medical conditions that could interfere with the study, or substance abuse or dependence within the past year. Healthy controls were included based on the following criteria: between 18 and 60 years old, matched for age, gender, race, education, height, weight, and body mass index (BMI) with MDD patients, a minimum of junior high school education, and right-handedness. Exclusion criteria for healthy controls included any DSM-IV Axis I psychiatric diagnosis, history of psychiatric illness, family history of psychiatric disorders, significant medical conditions that could interfere with the study, or substance abuse or dependence within the past year. The study adhered to the Declaration of Helsinki. The study protocol was approved by the Institutional Review Board of Wenzhou Seventh People’s Hospital (Approval No.: 2022-002). Informed consent was obtained from all participants. 40 healthy controls matched for age, gender, race, and education.

### Clinical Assessments

Initial assessments involved detailed clinical evaluations by two experienced psychiatrists to confirm DSM-IV MDD diagnoses. Baseline characteristics were recorded, including onset and diagnosis time, education, and medication history. For controls, similar demographic and educational information was gathered. All raters underwent standardized training, and inter-rater reliability was assessed with an intraclass correlation coefficient (ICC) exceeding 0.85 for consistency in clinical evaluations [31].

### NIRS Measurements

NIRS measurements were conducted using a 33-channel system, with 17 light emitters and 16 detectors fixed on a flexible cap positioned on the head of the participant. This system, with enhanced spatial resolution and comprehensive coverage of the prefrontal cortex, was crucial for capturing detailed hemodynamic changes associated with cognitive functions and MDD pathology [32]. Participants underwent NIRS while performing cognitive tasks designed to measure verbal fluency, sustained attention (e-primer task), and one-back memory. Continuous data on HbO and HbR levels in the prefrontal cortex were recorded during these tasks. Baseline NIRS data established normative spectra for the MDD and control groups. NIRS-Statistical Parametric Mapping (SPM) software (version 4.2, University College London, London, UK) was used to analyze the data, providing spectral, 2D, and 3D imaging results.

The NIRS testing environment was maintained with minimal noises, and participants were instructed to maintain stable emotional states and avoid unnecessary head movements during testing. The setup included a fixation point at eye level approximately one meter in front of the participant. The NIRS system used for this study included parameters such as Pre Scan (10 s), Wait Time (30 s), Stimulation (60 s), and Relax Time (70 s), with tasks involving verbal fluency, sustained attention, and one-back memory. The continuous analysis of hemoglobin data was configured with Base Start and End times set from –5 s to 0 s and a Moving Average period of 5.0 s. The data were processed for spatial mapping using the NIRS-SPM software to convert light-intensity data into hemoglobin concentration data.

### Treatment and Follow-up

During the 24-week treatment period, MDD patients received SSRIs such as fluoxetine, paroxetine, sertraline, or citalopram as monotherapy. In patients with severe sleep disturbances, short-term adjunctive treatment with zolpidem or eszopiclone was permitted. Clinical evaluations were conducted using the Hamilton Depression Scale [30] (HAMD) at baseline, 4 weeks, and 24 weeks. The HAMD assessed mood, guilt, suicide, insomnia, agitation, anxiety, weight loss, and somatic symptoms, with scores ranging from 0 to 52 (Cronbach’s alpha = 0.88). The Hamilton Anxiety Scale [33] (HAMA) evaluated mood, tension, fears, insomnia, intellectual impairment, depressed mood, somatic symptoms, and cardiovascular symptoms, with scores ranging from 0 to 56 (Cronbach’s alpha = 0.86). The Clinical Global Impression [34] (CGI) scale was also used, scoring from 1 (very much improved) to 7 (very much worse) (Cronbach’s alpha = 0.82). The Continuous Performance Test [35] (CPT) measured the number of correct responses, errors of omission, and errors of commission (Cronbach’s alpha = 0.85). The one-back tests [36] assessed the number of correct responses and reaction time (Cronbach’s alpha = 0.84). NIRS measurements were repeated at these time points to monitor hemodynamic changes and treatment response.

The specific dosage and usage instructions were as follows: fluoxetine (Eli Lilly and Company, Indianapolis, IN, USA; Batch No. 9902A): Start dose of 20 mg per day, which was increased to a maximum of 60 mg per day based on patient response and tolerance. Paroxetine (Zhejiang Huahai Pharmaceutical, Linhai, China; Batch No. 5301-17018M2): Start dose at 20 mg daily, with possible increases to 50 mg daily. Sertraline (Pfizer Inc., New York, NY, USA; Batch No. H10980141): Start dose at 50 mg daily, with possible increases to 200 mg daily. Citalopram (H. Lundbeck A/S, Copenhagen, Denmark; Batch No. V 4013): Start dose at 20 mg daily, with possible increases to 40 mg daily.

### Statistical Analysis 

Statistical analysis was performed using SPSS version 22.0 software (IBM Corp., Armonk, NY, USA). Data were first tested for normality using the Shapiro-Wilk test. Continuous data were expressed as mean ± standard deviation. Comparative analyses between groups were conducted using chi-square tests for categorical variables and independent *t*-tests for continuous variables. A *p*-value < 0.05 was considered statistically significant.

## Results

### Participant Characteristics

The demographic and baseline clinical characteristics of the participants are summarized in Table 1. There were no significant differences between the two groups in age, gender distribution, years of education, height, weight, or BMI (*p* > 0.05). As expected, the MDD group had significantly higher HAMD (*p* < 0.01) and HAMA (*p* < 0.01) scores compared to the control group.

**Table 1.** **Baseline characteristics for MDD patients and healthy controls**.

Characteristics	MDD group (n = 40)	Control group (n = 40)	χ^2^/t	*p*-value
Age (y)	37.75 ± 10.46	36.23 ± 9.83	0.67	0.51
Gender (male/female)	20/20	20/20	0.00	1.00
Education (y)	14.20 ± 3.22	14.40 ± 3.00	–0.29	0.77
Height (cm)	167.41 ± 5.73	168.27 ± 6.31	–0.64	0.53
Weight (kg)	65.34 ± 6.15	66.21 ± 6.23	–0.63	0.53
BMI (kg/m^2^)	23.14 ± 2.60	23.27 ± 2.35	–0.23	0.82
Onset age of MDD (y)	30.73 ± 9.04	-	-	-
Duration of illness (y)	5.76 ± 3.08	-	-	-
HAMD score	24.17 ± 3.35	3.08 ± 1.27	37.23	<0.01
HAMA score	20.42 ± 4.15	2.83 ± 1.15	25.83	<0.01

**Abbreviations**: MDD, major depressive disorder; BMI, body mass index; HAMD, Hamilton Depression Scale; HAMA, Hamilton Anxiety Scale. *p* < 0.05 represents statistical significance.

### Baseline NIRS Data

At baseline, significant differences were observed in the HbO and HbR levels between MDD patients and healthy controls. The MDD group showed reduced HbO and increased HbR in the prefrontal cortex during cognitive tasks. Specifically, MDD patients had significantly lower HbO levels (4.77 ± 1.23 µmol/L) compared to healthy controls (6.15 ± 1.01 µmol/L, *p* < 0.01), and significantly higher HbR levels (3.46 ± 0.98 µmol/L) compared to healthy controls (2.17 ± 0.85 µmol/L, *p* < 0.01) (Table 2).

**Table 2.** **Baseline NIRS Data for MDD patients and healthy controls**.

	MDD group (n = 40)	Control group (n = 40)	t	*p*-value
HbO (µmol/L)	4.77 ± 1.23	6.15 ± 1.01	5.48	<0.01
HbR (µmol/L)	3.46 ± 0.98	2.17 ± 0.85	6.29	<0.01

**Abbreviations**: NIRS, near-infrared spectroscopy; MDD, major depressive disorder; HbO, oxygenated hemoglobin; HbR, deoxygenated hemoglobin. *p* < 0.05 indicates statistical significance.

### NIRS Data at 4 Weeks and 24 Weeks

After 4 weeks and 24 weeks of treatment, significant improvements in HbO and reductions in HbR were observed in the MDD group, indicating enhanced cerebral hemodynamics. The healthy control group showed stable NIRS readings throughout the study period. By 4 weeks, HbO levels in MDD patients increased significantly from baseline (4.77 ± 1.23 µmol/L to 5.37 ± 1.21 µmol/L, *p* < 0.05) while HbR levels decreased significantly from baseline (3.46 ± 0.98 µmol/L to 2.91 ± 0.96 µmol/L, *p* < 0.05). However, at 4 weeks, the HbO levels in the MDD group were still significantly lower than those in the control group, and the HbR levels were significantly higher (*p* < 0.01). By 24 weeks, HbO levels in MDD patients increased (6.01 ± 1.08 µmol/L, *p* < 0.05), while HbR levels decreased (2.19 ± 0.71 µmol/L, *p* < 0.05), with no significant differences compared to the control group (*p* > 0.05). In the control group, no significant changes were observed in HbO or HbR levels (*p* > 0.05) (Table 3).

**Table 3.** **NIRS data for MDD patients and healthy controls**.

		MDD group (n = 40)	Control group (n = 40)	t	*p*-value
HbO (µmol/L)					
	Baseline	4.77 ± 1.23	6.15 ± 1.01	5.48	<0.01
	4 weeks	5.37 ± 1.21^*^	6.11 ± 1.10	2.86	<0.01
	24 weeks	6.01 ± 1.08^*^	6.16 ± 1.09	0.62	0.54
HbR (µmol/L)					
	Baseline	3.46 ± 0.98	2.17 ± 0.85	6.29	<0.01
	4 weeks	2.91 ± 0.96^*^	2.12 ± 0.78	4.17	<0.01
	24 weeks	2.19 ± 0.71^*^	2.10 ± 0.86	0.51	0.61

**Abbreviations**: NIRS, near-infrared spectroscopy; MDD, major depressive disorder; HbO, oxygenated hemoglobin; HbR, deoxygenated hemoglobin. *p* < 0.05 indicates statistical significance. ^*^p < 0.05 *vs*. baseline.

### Clinical Outcomes

Significant improvements in clinical outcomes were observed in the MDD group over the 24 weeks, as measured by the HAMD, HAMA, CGI, CPT, and one-back tests. The healthy control group maintained stable scores throughout the study.

At 4 weeks, the MDD group exhibited significant improvements in HAMD scores (24.17 ± 3.35 to 15.21 ± 4.08, *p* < 0.05), HAMA scores (20.42 ± 4.15 to 12.35 ± 3.45, *p* < 0.05), CGI scores (4.71 ± 0.81 to 3.14 ± 0.90, *p* < 0.05), CPT correct responses (84.25 ± 7.21 to 89.77 ± 5.78, *p* < 0.05), and one-back correct responses (78.50 ± 6.88 to 85.70 ± 5.37, *p* < 0.05). Despite these improvements, at 4 weeks, the HAMD, HAMA, and CGI scores in the MDD group remained higher than those in the control group (*p* < 0.01), while CPT and one-back correct responses were significantly lower than the control group (*p* < 0.01) (Table 4).

**Table 4.** **Clinical outcomes for MDD patients and healthy controls**.

		MDD group (n = 40)	Control group (n = 40)	t	*p*-value
HAMD score					
	Baseline	24.17 ± 3.35	3.08 ± 1.27	37.23	<0.01
	4 weeks	15.21 ± 4.08^*^	3.12 ± 1.25	17.92	<0.01
	24 weeks	7.63 ± 2.74^*^	3.03 ± 1.19	9.74	<0.01
HAMA score					
	Baseline	20.42 ± 4.15	2.83 ± 1.15	25.83	<0.01
	4 weeks	12.35 ± 3.45^*^	2.87 ± 1.10	16.56	<0.01
	24 weeks	5.90 ± 2.62^*^	2.77 ± 1.14	6.93	<0.01
CGI score					
	Baseline	4.71 ± 0.81	0.43 ± 0.15	32.86	<0.01
	4 weeks	3.14 ± 0.90^*^	0.42 ± 0.14	18.89	<0.01
	24 weeks	2.11 ± 0.42^*^	0.44 ± 0.13	24.02	<0.01
CPT					
	Baseline	84.25 ± 7.21	97.21 ± 3.72	10.10	<0.01
	4 weeks	89.77 ± 5.78^*^	96.47 ± 3.80	6.13	<0.01
	24 weeks	93.73 ± 4.24^*^	96.39 ± 3.73	2.98	<0.01
One-back					
	Baseline	78.50 ± 6.88	97.18 ± 2.71	15.98	<0.01
	4 weeks	85.70 ± 5.37^*^	97.59 ± 2.65	12.56	<0.01
	24 weeks	89.63 ± 4.32^*^	97.25 ± 2.53	9.63	<0.01

**Abbreviations**: MDD, major depressive disorder; HAMD, Hamilton Depression Scale; HAMA, Hamilton Anxiety Scale; CGI, Clinical Global Impression; CPT, Continuous Performance Test. ^*^p < 0.05 vs. Baseline.

At 24 weeks, further significant improvements were observed in the HAMD scores (15.21 ± 4.08 to 7.63 ± 2.74), HAMA scores (12.35 ± 3.45 to 5.90 ± 2.62), CGI scores (3.14 ± 0.90 to 2.11 ± 0.42), CPT correct responses (89.77 ± 5.78 to 93.73 ± 4.24), and one-back correct responses (85.70 ± 5.37 to 89.63 ± 4.32) in the MDD group (all *p* < 0.05). However, at 24 weeks, the MDD group still had higher HAMD, HAMA, and CGI scores than the control group (*p* < 0.01) and had significantly lower correct responses on the CPT and one-back tasks (*p* < 0.01). The healthy control group showed no significant changes in HAMD, HAMA, CGI, CPT, or one-back scores over the same period (*p* > 0.05) (Table 4).

## Discussion

The findings of this study demonstrate significant alterations in cerebral hemodynamics in MDD patients as measured by NIRS. At baseline, MDD patients exhibited significantly lower HbO levels and higher HbR levels in the prefrontal cortex compared to healthy controls. These results are consistent with previous studies that have identified hypofrontality, a condition characterized by reduced blood flow and oxygenation in the prefrontal cortex, as a hallmark of MDD [37, 38]. The prefrontal cortex is critical for executive functions, decision-making, and emotion regulation, and its impaired function is strongly associated with depressive symptoms [39].

The observed hemodynamic changes underscore the underlying neurobiological mechanisms of MDD. Reduced HbO levels suggest decreased neuronal activity and impaired neurovascular coupling, possibly contributing to the cognitive deficits and emotional dysregulation observed in MDD patients [40]. Conversely, elevated HbR levels indicate inefficient oxygen utilization, further exacerbating the functional impairment of the prefrontal cortex. This study confirms the potential of NIRS as a valuable tool for detecting these abnormalities, thus aiding in the early diagnosis of MDD and monitoring treatment response.

Our results align with other NIRS studies on MDD, which have similarly reported alterations in prefrontal hemodynamics during cognitive tasks [17, 21]. However, one study has reported mixed results regarding the extent and nature of these changes, possibly due to differences in sample characteristics, task paradigms, and NIRS methodologies. For example, variations in cognitive task design and duration influence the sensitivity of NIRS measurements, highlighting the need for standardized protocols in future research [41]. Our study utilized a comprehensive set of cognitive tasks, including verbal fluency, sustained attention, and one-back memory tests, contributing to the robust detection of hemodynamic changes.

The longitudinal design of our study reveals significant improvements in cerebral hemodynamics in MDD patients following 24 weeks of SSRI treatment. Elevations in HbO and declines in HbR levels were observed, indicating enhanced cerebral oxygenation and improved neurovascular function [40]. These changes correlate with clinical improvements in HAMD, HAMA, CGI, CPT, and one-back test scores, supporting the use of NIRS as an objective marker for treatment efficacy [42, 43]. These findings are significant, given the substantial variability in antidepressant responses. By providing early indicators of treatment response, NIRS can help tailor therapeutic strategies and reduce the duration of ineffective treatments. Moreover, a 24-week period allows for the observation of both short-term and sustained effects of treatment, providing a comprehensive view of the treatment trajectory [44]. This duration also aligns with clinical guidelines that recommend a continuation phase of treatment lasting several months to consolidate response and prevent relapse [45].

However, it is important to acknowledge potential confounding factors that could influence our results, especially medication adherence and lifestyle factors. Medication adherence significantly affects treatment outcomes, as inconsistent use of prescribed medication leads to suboptimal therapeutic effects [46]. Although we encouraged adherence through regular follow-ups, the reliance on self-reported adherence introduces variability. Future studies should use objective measures such as pill counts or electronic monitoring to ensure accurate adherence tracking [47]. Additionally, lifestyle factors such as diet, exercise, and sleep patterns impact MDD symptoms and treatment response [48]. While we collected baseline lifestyle information, continuous and detailed monitoring of these factors was beyond the scope of this study. Incorporating comprehensive lifestyle assessments in future research will help control for these potential confounders and provide clearer insights into the treatment effects.

Despite the promising results, this study has several limitations. The sample size, while adequate for detecting significant differences, may limit the generalizability of the findings. Larger, multi-center studies are warranted to validate our findings and establish normative NIRS data across diverse populations. Additionally, while our study focused on the prefrontal cortex, other brain regions implicated in MDD, such as the amygdala and hippocampus, should be examined using advanced NIRS techniques or complementary imaging modalities [49]. Furthermore, the potential confounding effects of medication adherence, lifestyle factors, and comorbid conditions are not fully controlled in this study and warrant further investigation.

## Conclusion

In conclusion, this study underscores the utility of NIRS in detecting and monitoring cerebral hemodynamic changes in MDD. The significant improvements in HbO and HbR levels following SSRI treatment and corresponding clinical improvements highlight the potential of NIRS as a non-invasive, objective tool for assessing treatment efficacy. Our findings contribute to the growing body of evidence supporting the integration of neuroimaging biomarkers into clinical practice, offering a path toward more personalized and effective treatment strategies for MDD. Future research should refine NIRS methodologies, explore additional biomarkers, and expand the scope of neuroimaging studies to include larger, more diverse cohorts. Addressing these challenges will enhance our understanding of the neurobiological underpinnings of MDD and improve outcomes for patients suffering from this debilitating disorder.

## Availability of Data and Materials

The data used to support the findings of this study are available from the corresponding author upon request.

## Supplementary Material

Supplementary material associated with this article can be found, in the online version, at https://doi.org/10.62641/aep.v53i2.1708.
