# Supplementary material for: Application of Near-Infrared Spectroscopy in Early Detection of Antidepressant Treatment Efficacy in Major Depressive Disorder: A Longitudinal Study
Source: Actas Esp Psiquiatr. 2025 Mar 5;53(2):275–83. doi: 10.62641/aep.v53i2.1708 (PMC11898260; doi:10.62641/aep.v53i2.1708)
Supplement: Supplementary file 1 [file ActEsp-53-2-275-283-s1.docx]

Supplementary Table 1. Baseline characteristics for MDD patients and healthy controls (n = 138).

|  | Control group (n=75) | MDD group (n=63) | χ^2^/t | *p* value |
| --- | --- | --- | --- | --- |
| Age, years (mean ± SD) | 32.81 ± 10.53 | 40.23 ± 12.03 | 3.863 | <0.001 |
| Gender (%) |  |  | 23.867 | <0.001 |
| male | 55 (73.3) | 20 (31.7) |  |  |
| female | 20 (26.7) | 43 (68.3) |  |  |
| Education, years (mean ± SD) | 14.41 ± 3.01 | 14.25 ± 3.26 | 0.300 | 0.765 |
| Height, cm (mean ± SD) | 168.71 ± 6.13 | 168.00 ± 5.60 | 0.705 | 0.482 |
| Weight, kg (mean ± SD) | 65.71 ± 6.22 | 65.06 ± 6.17 | 0.614 | 0.540 |
| BMI, kg/m^2^ (mean ± SD) | 23.57 ± 2.12 | 23.08 ± 2.58 | 1.225 | 0.223 |
